# Testing for consistency in the impacts of a burrowing ecosystem engineer on soil and vegetation characteristics across biomes

**DOI:** 10.1038/s41598-019-55917-x

**Published:** 2019-12-18

**Authors:** M. A. Louw, N. S. Haussmann, P. C. le Roux

**Affiliations:** 1 Department of Plant and Soil Sciences, University of Pretoria, Pretoria, Private Bag X20, Hatfield, 0028 South Africa; 2 Department of Geography, Geoinformatics and Meteorology, University of Pretoria, Pretoria, Private Bag X20, Hatfield, 0028 South Africa; 3 Department of Biological Sciences, University of Cape Town, Private Bag X3, Rondebosch, 7701 South Africa; 4 Centre for Statistics in Ecology, Environment and Conservation, Department of Statistical Sciences, University of Cape Town, Private Bag X3, Rondebosch, 7701 South Africa

**Keywords:** Community ecology, Plant ecology

## Abstract

The impacts of ecosystem engineers may be expected to vary along environmental gradients. Due to some resources being more limited in arid than in mesic environments, disturbances created by burrowing mammals are expected to have a greater ameliorating effect in arid environments, with larger differences in microhabitat conditions expected between burrows and undisturbed areas. The aim of this study was to test if the impacts of a medium-sized burrowing mammal, the aardvark, on soil properties (soil temperature, moisture and compaction) and vegetation characteristics (plant cover, species richness and species composition) are consistent across three biomes that differ strongly in annual rainfall. Burrowing affected soil and vegetation attributes, but the direction and magnitude of these biogeomorphological impacts were not consistent across the different biomes. For example, plant species composition was altered by burrowing in the arid scrubland and in the mesic grassland, but not in the semi-arid savannah. Contrary to expectations, the difference in the impacts of burrowing between biomes were not related to rainfall, with burrowing having strong, albeit different, impacts in both the arid scrubland and the mesic grassland, but weaker effects in the semi-arid savannah. It appears, therefore, that the impacts of these biogeomorphic agents may be site-specific and that it may be difficult to predict variation in their biotic and abiotic effects across environmental gradients. As a result, forecasting the impacts of ecosystem engineers under different conditions remains a challenge to management, restoration and conservation strategies related to these types of species.

## Introduction

Ecosystem engineers affect ecosystem structure and functioning by creating, modifying, maintaining and destroying habitats through altering the distribution of resources in the environment^1–3^. By creating patches of unique habitat differing from ambient conditions, ecosystem engineers can mediate the response of species (and communities) to environmental conditions^3,4^, enabling, for example, species to occur in habitats that would otherwise not be suitable^5^. Therefore, ecosystem engineers influence the distribution and abundance of species^3,6,7^ and thereby alter community characteristics^3^.

Ecosystem engineers generally have a positive impact on species richness at the landscape-level by increasing habitat diversity^2,6^, and the loss or introduction of such species may disproportionally affect the distribution of other organisms and the functioning of ecosystems^8^. However, the nature and strength of the impact of an ecosystem engineer may vary spatio-temporally, depending on environmental conditions, community characteristics and the resources altered by the engineer^8,9^. For example, herbaceous species richness in beaver meadows (i.e. wetlands formed by beavers damming streams) varies depending on site hydrology, with higher richness in areas of more variable water table depth and faster drainage^10^. As a result, a specific ecosystem engineer can potentially have very different species- and community-level impacts in different environments, even when the engineer functions similarly in all environments^8^. It is therefore challenging to predict when and where ecosystem engineers will have a strong impact on communities and ecosystem processes, despite this being an important question in ecology^4,9^.

One of the mechanisms through which ecosystem engineers interact with their environment, and specifically increase environmental heterogeneity, is through creating local disturbances^11^. Burrowing mammals, in particular, can be important agents of disturbance as they can displace large volumes of soil, thereby, creating discrete habitat patches, and potentially constructing new landforms, within landscapes at relatively small spatial scales^12–18^. Burrowing can result in large geomorphic changes in the landscape by destabilizing soils and enhancing erosion^19^, possibly creating geomorphic signatures in the landscape^20^. As a result, burrowing mammals are often considered to be both biogeomorphic agents^18,21^ and ecosystem engineers^13,22^. Indeed, in this context there is considerable overlap between these two concepts, since the formation and decay of burrows (i.e. reflecting the landform-focused approach of biogeomorphology) has consequences for species occurring in the landscape (i.e. in line with the organism-focus of ecosystem engineering)^21^.

Through burrowing, these animals contribute to soil mixing and the creation of soil patches that differ chemically and physically from surrounding soils^12,13,23,24^. Burrow soils generally have a higher nutrient concentration than undisturbed surrounding soils^25^, with higher enzyme activity^26^ and enhanced mineralization rates^27^. As a result, these soils may contain a higher inorganic nitrogen content, which is available to plants and promotes growth^27,28^. Burrows also often enhance water infiltration and drainage^29,30^, thereby reducing run-off^31^. Furthermore, burrow mound soils are usually less compacted than surrounding undisturbed soils^32,33^. By decreasing soil compaction there may be an increase in water availability, aeration and root space in soil, thereby enhancing water and nutrient uptake by plants^34^. Burrows typically also provide a more favourable microclimate than ambient conditions by buffering thermal extremes^35–39^. Thus, the activities of burrowing mammals may considerably alter soil properties and create new microhabitats which may preferentially be utilized by other organisms within the landscape.

Due to burrowing altering soil conditions, burrows generally support different plant communities than surrounding undisturbed areas^12^. Often a higher number of pioneer species^40^, short-lived species^25,35^ and/or alien species may be found at burrows than in undisturbed surrounding areas^41^. Furthermore, burrowing lowers plant density locally, potentially reducing competition between plants^24,42^, and facilitating their co-existence^42,43^. In addition, seed germination and seedling establishment is often enhanced relative to adjacent substrates due to altered microclimatic conditions within burrows^16,44^. These impacts of burrowing can allow plant species that did not previously occur in the surrounding undisturbed areas to establish^45^ and may potentially increase plant species richness at a local scale^40,42^. Burrowing mammals can therefore strongly affect plant community composition through biogeomorphological disturbances by creating new microhabitats that enhance the establishment, growth and survival of some plant species.

However, burrowing also sometimes affects plants negatively, as evident at newly created or recently abandoned burrows where plant cover is often greatly reduced relative to adjacent undisturbed areas^46–48^. For example, the disturbances associated with excavation may reduce plant species richness immediately around burrows^47,49^. As burrows age, their plant cover and species richness generally increase^46^. Indeed, plant cover and species richness at long-abandoned burrows is usually higher than at newly created burrows, and may even be higher than in undisturbed areas^48^. Therefore the age of burrows and excavated soil mounds could mediate their influence on vegetation characteristics with new and old soil disturbances potentially having contrasting effects on plant species and their cover, and possibly even contributing to a shifting mosaic of microhabitat conditions and plant communities within a landscape^43^.

The widespread distribution of some ecosystem engineer species provide an opportunity to test for the consistency of their impacts in a range of habitat types. The biogeomorphological impacts of ecosystem engineers are expected to be greater in harsh environments, including drylands^26,31,50^. In drylands, resources are generally more limiting, through both time and space, than in more mesic environments^31,51^. One would therefore expect that burrowing mammals will have the strongest effects on soil properties in arid and semi-arid areas, where the accumulation of resources results in patches that differ more greatly from undisturbed areas than in mesic environments^31^. Therefore, the influence of soil disturbances by burrowing mammals on ameliorating habitat conditions and concentrating resources is expected to benefit plants more in arid habitats than in mesic environments.

The aim of this study was, therefore, to test whether the biogeomorphological impacts of burrowing by medium-sized mammals, chiefly aardvark (*Orycteropus afer*), differ across three environments, and whether these effects are mediated by burrow age. To determine this, we examined the impact of burrowing on physical soil properties (soil temperature, moisture and compaction) and vegetation properties (vegetation cover, species richness and vegetation composition) for three different burrow ages (fresh, abandoned and collapsed) at three sites differing considerably in annual precipitation (170–720 mm p.a.) and vegetation type (arid scrubland, semi-arid savannah and mesic grassland).

## Materials and Methods

### Study sites

**Figure 1:**
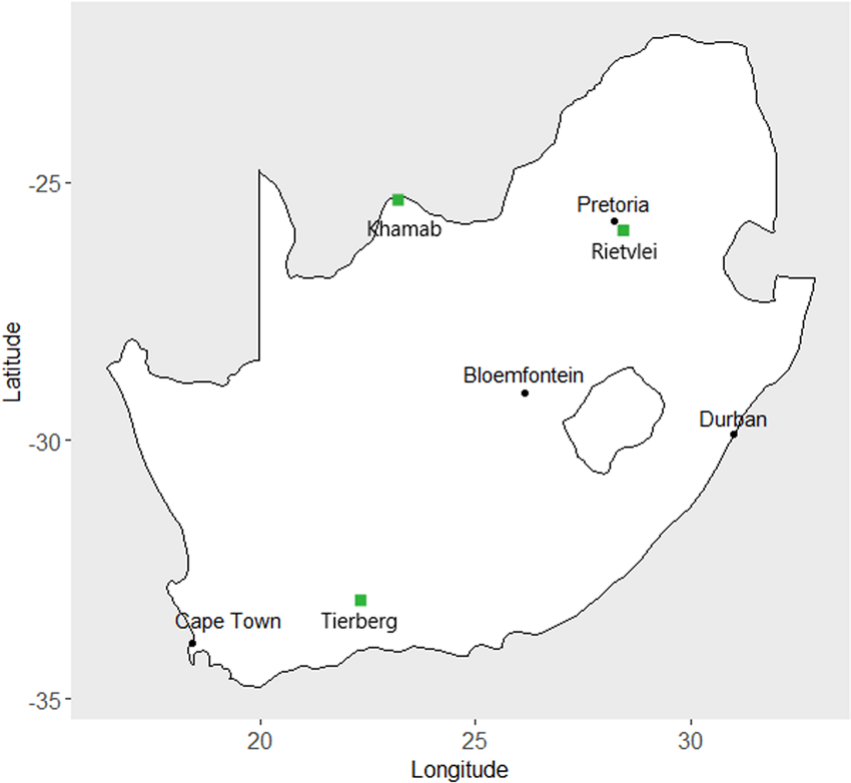
Location of study sites in South Africa.

Three sites were selected in three different biomes in South Africa, spanning a range of annual rainfall: Rietvlei Nature Reserve (c. 720 mm per annum; mesic grassland), Khamab Kalahari Reserve (c. 330 mm per annum; semi-arid savannah) and Tierberg Karoo Research Centre (c. 170 mm per annum; arid scrubland at the border of Succulent- and Nama Karoo, Fig. 1) (see ref. ^52^ for details on these biomes). Data from the arid scrubland and mesic grassland have previously been reported in a different context (see ref. ^53,54^). Aardvark occur at all three sites and are the dominant medium-sized (i.e. c. 40–60 kg) burrowers across all sites (R. Marais; S. Milton; H. Kilian pers. comm.).

### Burrow selection and classification

In the mesic grassland, burrows were selected from an extensive burrow survey performed during April 2015. Randomly selected 1 ha plots within all reserve management blocks were searched for burrows, with 60 of the resulting 203 burrows randomly selected for sampling during April and May 2015^54^. In the arid scrubland, burrows were located systematically during April 2015 by traversing the study site. Eleven burrows were found across the entire reserve, but one was occupied by a beehive and no soil measurements could be obtained from it^53^. In the semi-arid savannah, five adjacent reserve management blocks within the southern part of the reserve were selected during March 2016 and burrows were located opportunistically within these management blocks (a total of 71 burrows were sampled). At all sites, burrows had a tunnel shape structure with a roof, distinguishing them from shallow feeding scrapes and natural depressions. In addition, all sampled burrows were separated by at least 10 m.

As an indication of time that had passed since burrowing ceased, a distinction was made between recently abandoned or currently used burrows (hereafter “fresh” burrows), abandoned burrows, and collapsed burrows (presumed to have been deserted for a longer period than abandoned burrows, see Supplementary Fig. S.1). Hereafter, “time since burrowing ceased” will be referred to as “burrow age” for simplicity (i.e. at older burrows, burrowing would have ceased for a longer period than at fresh burrows). In the arid scrubland, data from abandoned and collapsed burrows were combined due to the small sample size for collapsed burrows. Although none of the burrows were occupied during sampling, it is possible that some of the fresh burrows were still sporadically used by medium-sized mammals.

### Data collection

Data were gathered at three microsites at each burrow: (1) at the burrow entrance, (2) at the impacted area around the burrow (i.e. disturbed by the deposition of excavated soil), and (3) at a control site close to the burrow. In the mesic grassland and the semi-arid savannah, the impacted area was the area opposite the burrow entrance where soil was displaced to by the burrower (i.e. the excavated soil mound). In the arid scrubland burrows were exclusively located on heuweltjies (i.e. aardvark burrowing was limited to nutrient-rich earth mounds)^53^, and it was difficult to distinguish heuweltjie soil from excavated burrow soil. Thus, the impacted area was considered as the whole heuweltjie around the burrow, with the control taken as the nearest heuweltjie that showed no signs of medium-sized mammal burrowing (i.e. in the arid scrubland all sampling was conducted on heuweltjies). In the mesic and the semi-arid sites, the control area for each burrow was chosen two meters away from the burrow at a 90° angle to the orientation of the burrow tunnel (to avoid sampling the surface potentially above the burrow tunnel), where there were no visible signs of burrowing disturbance. At all sites the burrow entrance measurements were taken below the edge of the tunnel roof, avoiding heavily shaded areas deeper within the burrow where plants are unlikely to establish due to a lack of solar radiation.

In the mesic grassland and the semi-arid savannah a quadrat with a surface area of 0.25 m^2^ was placed over each microsite. This quadrat size was chosen to accommodate measurements at the burrow entrances, which were mostly narrow. All soil measurements were taken within this quadrat. In the arid scrubland, a quadrat of 0.25 m^2^ was also sampled at the burrow entrance. However, as entire heuweltjies were sampled for both the control and the impacted areas in the arid scrubland, the sample area here differed between burrows. Although, heuweltjies with and without burrows did not differ significantly in size (ANOVA: F = 0.005, p > 0.05), covering on average 62 m^2^, the difference in sample area between the burrow entrance and the other two microsites meant that differences in species richness between microsites in the arid scrubland had to be interpreted cautiously (since species richness is an area-dependent metric, and is therefore underestimated in the burrow entrances relative to the other two microsites in the arid scrubland). However, the soil and other vegetation characteristics are not dependent on sample area and still provide for robust comparisons between microsites in that study site.

At each microsite all vascular plants were identified to species level where possible, and canopy cover for each species was visually estimated. Three soil properties were also measured at each microsite: (1) volumetric water content within the top 3.8 cm of the soil (TDR 300 soil moisture meter; Spectrum technologies; USA), (2) soil resistance to penetration at 5 mm depth (as a measure of soil compaction) using a hand-held pocket penetrometer (Geotest; USA), and (3) soil temperature at 2 cm depth using a hand-held RTD thermometer (Eutech Instruments; RSA).

### Statistical analyses

Data were analysed using linear mixed effect models for variables with a normal distribution (soil moisture and temperature) and generalized linear mixed effect models (GLMMs) for variables that were not normally distributed (soil compaction, vegetation cover and species richness). These analyses were implemented using the lme4 package^55^ and the MASS package^56^ in R statistical software^57^. Microsites (i.e. burrow entrance, impacted area and control) and burrow age (i.e. fresh, abandoned and collapsed) were set as fixed effects. The location of each burrow and its associated microsites were set as a random effect to account for potential differences in environmental conditions across each study area. Since the impact of burrows on soil temperature may differ between warmer and cooler times of the day^38^, the interaction of time and microsite was included as a predictor variable. Soil compaction data were analysed assuming a binomial distribution since the penetrometer data was bounded at both its upper and lower limits. Vegetation cover and species richness data were analysed assuming a binomial and a Poisson distribution respectively. The interaction between microsite and burrow age was included in models when it significantly improved model fit (assessed using the *anova* function). However, for models that required analysis using the *glmmPQL* function (due to algorithm failure with the *glmer* function), formal model comparisons were not possible. In such analyses the more complex model (i.e. including the interaction term) was only reported when the interaction term’s coefficients were significantly different from zero.

Non-metric Multidimensional Scaling ordination and Permutational Multivariate Analysis of Variance^58^ were used to test for differences in species composition between burrow entrances, impacted areas and controls (implemented using the vegan package^59^). The effect of the interaction between microsite and burrow age was tested, but was never significant and is therefore not reported. Chi^2^ tests were used to determine whether common species (occurring > 3 times in a dataset) were non-randomly distributed across the different microsites. In the arid site burrow entrances were excluded from Chi^2^ tests due to differences in sample area.

## Results

**Figure 2:**
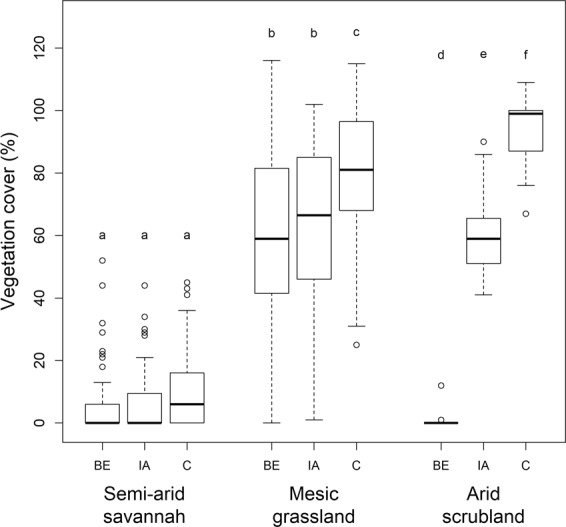
Vegetation cover at different microsites at each study site. BE = “Burrow entrance”, IA = “Impacted area”, C = “Control”. Microsites within a study site not sharing a letter differ significantly. Plots indicate median values (thick lines), interquartile range and range (box and whiskers, respectively), and outliers (empty circles).

Vegetation cover differed significantly between microsites in both the mesic grassland and the arid scrubland (ANOVA: mesic grassland: Chi^2^ = 17.01, p < 0.001, arid scrubland: Chi^2^ = 56.20, p < 0.001), but not at the semi-arid savannah (Chi^2^ = 0.99, p > 0.1, Table 1, Fig. 2, see Supplementary Table S.1 for further details). Furthermore, there was significantly higher vegetation cover at collapsed burrows than at fresh burrows in the semi-arid savannah site (Chi^2^ = 7.71, p < 0.05), and a marginally significant difference between the burrow age classes in the mesic grassland (Chi^2^ = 5.62, p = 0.06, Supplementary Table S.1).

**Table 1:** Summary of burrowing impacts on soil and vegetation in comparison to undisturbed control areas in three different biomes.

Study site	Microsite	Vegetation cover	Species richness	Soil temperature	Soil moisture	Soil compaction
Mesic grassland	BE	**↓**	**↑**	**↓**	**↓**	**↓**
	IA	**↓**	⇩	**↑**	**↓**	**↓**
Semi-arid savannah	BE	⇩	**↓**	**↓**	**↑**	**↓**
	IA	⇩	**↓**	Weak effect	**↑**	**↑**
Arid scrubland	BE	**↓**	**↓**	Buffer	⇧	No effect
	IA	**↓**	⇩	Weak effect	⇧	⇧

BE = “Burrow entrance”, IA = “Impacted area”. ↑ =  increase, ↓ = decrease. Filled arrows indicate significant differences, empty arrows indicate non-significant differences.

**Figure 3:**
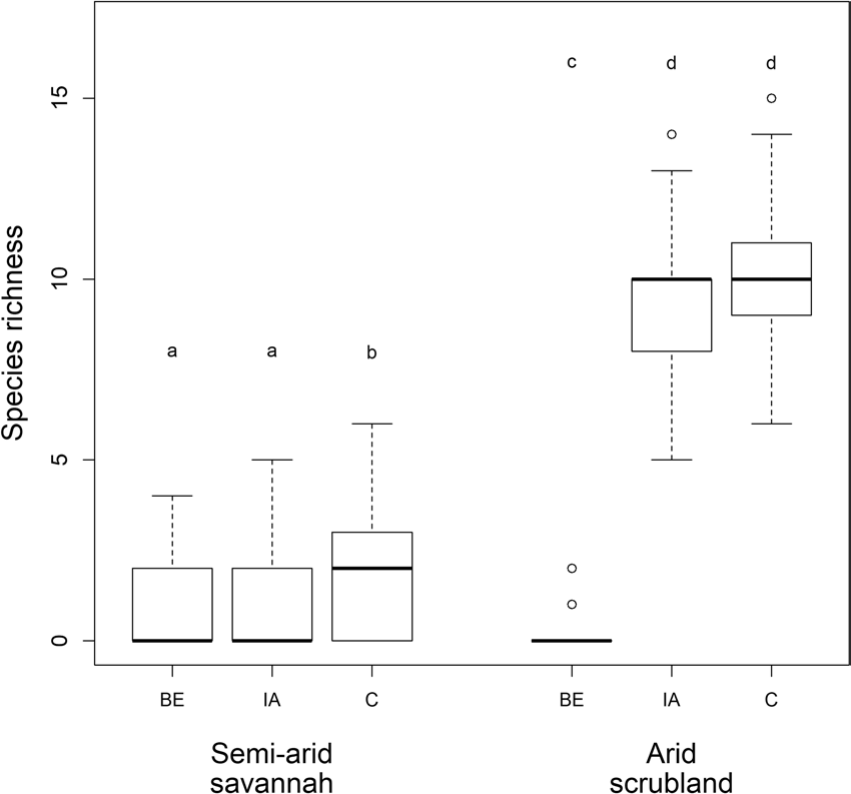
Species richness at the different microsites at two study sites. BE = “Burrow entrance”, IA = “Impacted area”, C = “Control”. Microsites within a site not sharing a letter differ significantly. Plots indicate median values (thick lines), interquartile range and range (box and whiskers, respectively), and outliers (empty circles). See Fig. 4 for results for the mesic grassland site, and Supplementary Table S.1 for statistical results. Note that at the arid scrubland sample area differed between the burrow entrance and the other two microsites.

**Figure 4:**
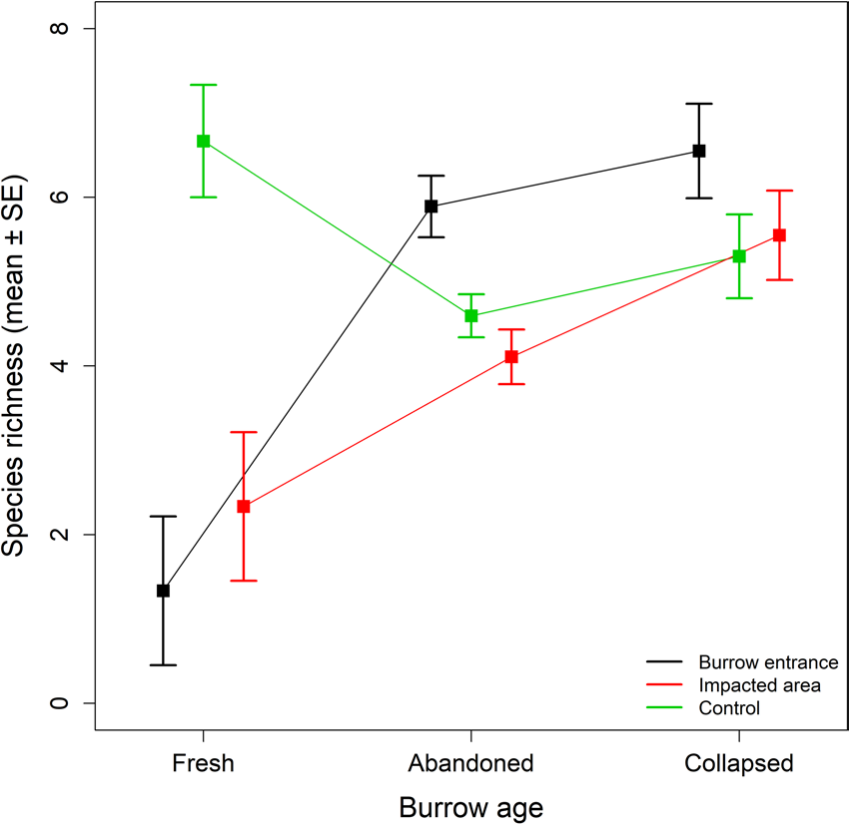
Species richness at the mesic grassland in different microsites for different burrow age classes. See Fig. 3 for results for the semi-arid savannah and the arid scrubland site, and Supplementary Table S.1 for statistical results.

Species richness differed significantly between the microsites at all sites (ANOVA: semi-arid savannah: Chi^2^ = 19.54, p < 0.001, arid scrubland: Chi^2^ = 38.49, p < 0.001, Fig. 3, Table 1, Supplementary Table S.1). Moreover, in the mesic grassland species richness differed significantly both between microsites (Chi^2^ = 12.79, p < 0.01) and burrow age classes (Chi^2^ = 6.02, p < 0.05), with the impact of microsite depending significantly on burrow age (interaction term: Chi^2^ = 15.01, p < 0.01, Fig. 4, Supplementary Table S.1). In addition, in the semi-arid savannah species richness was significantly lower at the fresh burrows than at the other burrow age classes (Chi^2^ = 18.87, p < 0.001, Supplementary Table S.1).

A total of 166 species were recorded in the mesic grassland, 32 in the semi-arid savannah and 30 in the arid scrubland. Some common species (i.e. occurring > 3 times in a dataset) were non-randomly distributed between microsites (c. 20% of species in semi-arid savannah and the mesic grassland, but 0% in arid scrubland, Supplementary Tables S.4–S.6). However, of these species only two occurred exclusively at a specific microsite, with the fern species *Cheilanthes viridis* and *Pellaea calomelanos*, occurring exclusively at burrow entrances in the mesic grassland (see Supplementary Table S.5 for details).

There were clear differences in species composition between the microsites in the arid scrubland, and there were small (but statistically significant) differences in composition between microsites in the mesic grassland (Table 2, Supplementary Fig. S.2). Burrow age also significantly affected species composition in the mesic grassland and in the semi-arid savannah (Table 2, Supplementary Fig. S.2), albeit weakly.

**Table 2:** Effects of burrowing mammals on vegetation composition at different study sites.

Response variable	Study site	Fixed effect	F	R^2^ (%)	P
Composition	Mesic grassland	Microsites	4.14	4.54	***
		Age	1.73	1.90	*
	Semi-arid savannah	Microsites	0.99	1.77	
		Age	1.82	3.23	*
	Arid scrubland	Microsites	3.22	24.36	**
		Age	1.00	3.79	

*p < 0.05, **p < 0.01, ***p < 0.001.

**Figure 5:**
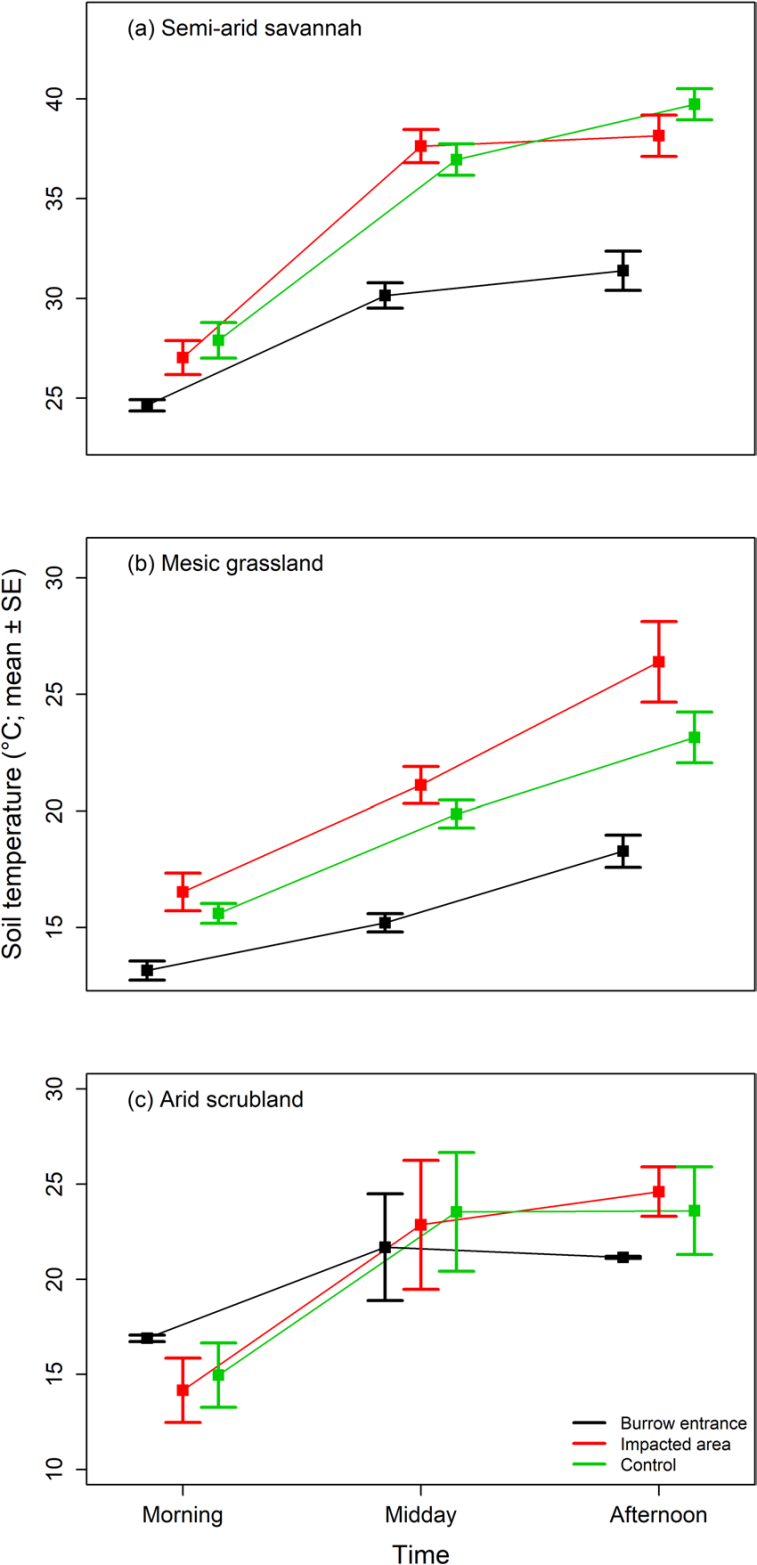
Soil temperature of microsites at three different times of day at the different study sites. Morning: 7 h 15–10 h 05, Midday: 10 h 05–12 h 10, Afternoon: 12 h 10–15 h 45.

Soil temperature differed significantly between the microsites, with nature and magnitude of the temperature differences depending on the time of day at all of the study sites (ANOVA: mesic grassland: Chi^2^ = 23.80, p < 0.001, semi-arid savannah: Chi^2^ = 21.57, p < 0.001, arid scrubland: Chi^2^ = 21.30, p < 0.001, Fig. 5, Table 1, Supplementary Table S.7). In the mesic grassland and the semi-arid savannah burrow entrances were always cooler than the other microsites, although these differences were smaller during the cooler part of the day. In the arid scrubland burrows, soil temperatures were buffered, being cooler than the rest of the microsites during the warmest times of day, but warmer during the cooler part of the day.

**Figure 6:**
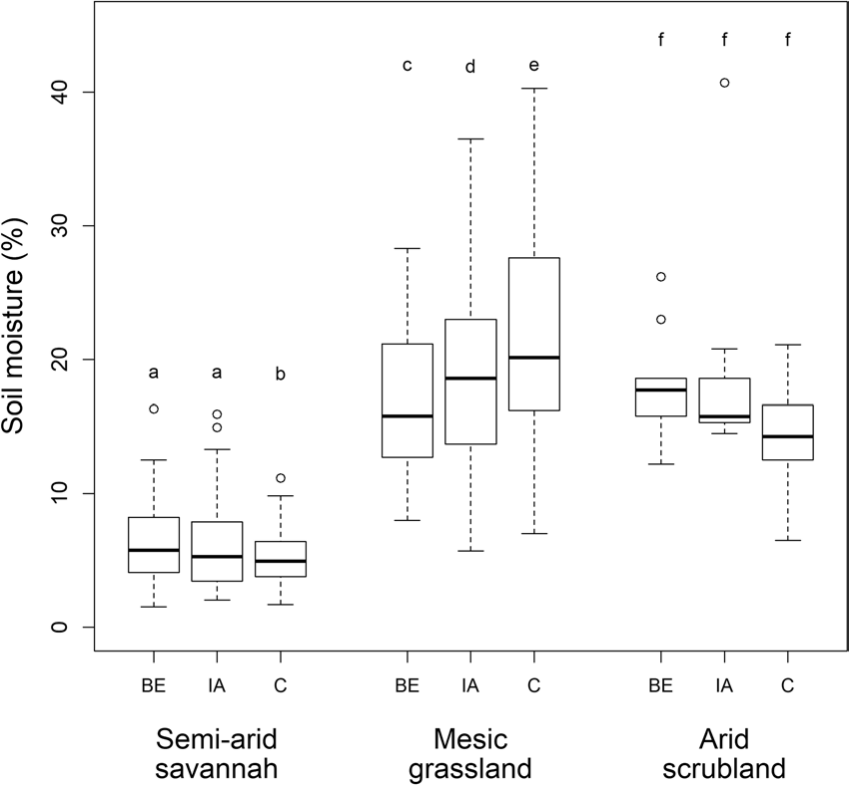
Soil moisture (measured as volumetric water content) of microsites at the different study areas. BE = “Burrow entrance”, IA = “Impacted Area”, C = “Control”. Microsites within a study site not sharing a letter differ significantly. Plots indicate median values (thick lines), interquartile range and range (box and whiskers, respectively), and outliers (empty circles).

In the mesic grassland soil moisture was highest in the control plots (ANOVA: Chi^2^ = 51.31, p < 0.001), while the opposite occurred in the semi-arid savannah (Chi^2^ = 23.03, p < 0.001, Fig. 6, Table 1, Supplementary Table S.7). In the arid scrubland there were no significant differences in soil moisture between the different microsites (Chi^2^ = 4.34, p > 0.05, Fig. 6, Table 1, Supplementary Table S.7).

**Figure 7:**
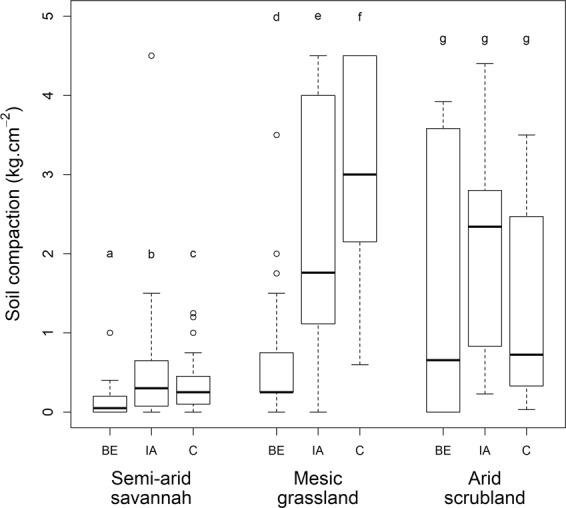
Soil compaction of microsites at the different study areas. BE = “Burrow entrance”, IA = “Impacted Area”, C = “Control”. Microsites within a study site not sharing a letter differ significantly. Plots indicate median values (thick lines), interquartile range and range (box and whiskers, respectively), and outliers (empty circles).

Burrowing lowered soil compaction significantly in both the mesic grassland (ANOVA: Chi^2^ = 25.62, p < 0.001) and the semi-arid savannah (Chi^2^ = 64.85, p < 0.001, Fig. 7, Table 1, Supplementary Table S.7). Impacted area soils were also less compact than soils at the control plots in the mesic grassland, but were more compact in the semi-arid savannah. In contrast, in the arid scrubland there were no significant differences in soil compaction between the microsites (Chi^2^ = 2.40, p > 0.1, Fig. 7, Table 1, Supplementary Table S.7). Burrow age class did not significantly affect soil temperature, moisture or compaction at any of the study sites (Supplementary Table S.7).

## Discussion

Burrowing affected vegetation characteristics and soil properties in all three environments. These biogeomorphological impacts were mostly inconsistent between the sites, differing in their nature and/or magnitude, and highlight the difficulty of predicting the impacts of ecosystem engineering in different environments. Some impacts were, however, consistent across all three sites, including lower vegetation cover at both burrow entrances and impacted areas than at controls. This is consistent with results from other studies, which show lower plant cover at burrow mounds than in undisturbed surrounding areas^41,47^, and in some cases even at mounds of long abandoned burrows^45^. In our study, the lower plant cover is not driven by changes to soil physical properties (i.e. soil temperature, moisture and compaction) within and around burrows, because burrowing had inconsistent effects on soil characteristics at the different sites. The reduced cover is therefore, more likely due to the burying and uprooting of vegetation during burrow creation^60,61^. Hereafter, vegetation cover may remain low at burrows for an extended time, possibly due to disturbances associated with the use of burrows by both the original creator and other subsequent occupant species. As a result, disturbances associated with the creation of burrows may negatively affect vegetation cover, potentially for the entire lifespan of the burrow^45^.

Burrowing decreased plant species richness, except at burrow entrances in the mesic grassland where species richness was higher than the other microsites at both abandoned and collapsed burrows. This suggests that burrowing increased habitat diversity and/or opportunities for establishment for plant species in the mesic site^40,48^. It is possible that the substantially lower soil compaction here facilitated the establishment of plant species^62^. Less compact soils at burrow entrances will be more aerated^28^ and may have higher water infiltration rates^63^, which may also explain the drier soil conditions found at this microsite in the mesic grassland^64^. Although not considered in this study, variation in the effect of burrowing on soil moisture may also be driven by fine-scale heterogeneity within and between the three study sites (e.g. burrow orientation and slope, humus content of soil and time since rainfall^65,66^).

Burrowing mammals are known to affect vegetation composition in a variety of vegetation types and under diverse climatic conditions. For example, different plant communities exist at burrows relative to undisturbed areas in temperate forests^42^, low-alpine mountain tundra^49^, semi-arid savannah, Nama Karoo, Albany thicket^45^, open woodland^41^, Mediterranean dehesa^67^, and *Stipa* steppe^47^. In this study, burrowing affected species composition strongly in the arid scrubland, with a weaker impact on species composition in the mesic grassland. The strong impact of burrowing on vegetation composition in the arid scrubland is probably due to burrow entrances being largely unvegetated (and not due to turnover in species composition). However, burrowing does not always affect vegetation composition^68^, and in the semi-arid site vegetation composition was similar at burrows and in undisturbed areas. Therefore, similar to its impacts on species richness, burrowing did not have a consistent effect on species composition across the three environments.

A fairly low percentage of common plants were non-randomly distributed between microsites. This is probably due to the seeds of plants colonising the burrows chiefly coming from the undisturbed control areas (i.e. due to all microsites sharing the same potential species pool)^68,69^. Moreover, although burrowing altered soil properties, it probably did not alter soil to such a great extent that plants from surrounding areas were entirely unable to establish. Indeed, only two plant species were unique to any microsites, with the fern species, *Cheilanthes viridis* and *Pellaea calomelanos*, occurring exclusively at burrow entrances in the mesic grassland. Burrows could have facilitated the survival of these ferns as both species are weak competitors^70^ and prefer drier habitats^71^.

The local-scale impact of burrowing on species cover and composition may potentially also have impacts on other taxa, including pollinators and herbivores. For example, in the mesic site unpalatable invasive plant species, including *Verbena bonariensis* and *Tagetes minuta*, occurred significantly more frequently at burrow entrances. In addition, *Eragrostis chloromelas*, a climax grass with low palatability^72^, occurred less frequently at burrows, while *Pollichia campestris* a pioneer species which is favoured by browsing animals^73^ occurred more frequently at these same microsites in the mesic site. In the semi-arid savannah *Schmidtia pappophoroides* (a highly palatable perennial climax grass species^74^) and *Acanthosicyos naudinianus* (an important source of moisture for mammalian herbivores^75^) both occurred significantly less frequently in burrowed sites. Therefore, while not explicitly considered in this study, through impacts on vegetation, burrowing may possibly also have knock-on effects on other trophic levels, especially in ecosystems where burrowing animals occur at high densities and/or burrowed microsites differ most in abiotic conditions from unburrowed areas.

While not as important as microsite, burrow age also affected some vegetation properties. Fresh burrows supported simpler plant communities, with lower species richness and vegetation cover, than older burrows in the semi-arid savannah and in the mesic grassland. This likely reflects that at fresh burrows disturbances only recently ceased (or were possibly still occurring), and that few species were able to withstand the associated mechanical damage. Once burrowing (and all associated disturbances) stops, more species are able to establish and burrows start providing microhabitat benefits, reflected in species richness increasing after abandonment^48^. Therefore, at fresh burrows the negative effects of constant disturbances (e.g. uprooting and burial) outweigh the positives provided by the burrows (e.g. through reduced competition and/or altered microclimatic conditions)^45^. As a result, the vegetation composition of abandoned burrows is expected to gradually become more similar to surrounding undisturbed vegetation^47^, although, in some cases, as burrows get older differences in vegetation composition may persist^45,46^. These changes in vegetation properties through time are probably not related to changing abiotic conditions as soil properties was not related to burrow age. In contrast, changes in vegetation with burrow age likely reflects successional processes^48^, driven by differential colonization rates of different plant groups^46^. Burrowing therefore, through small-scale biogemorphological disturbances, appears to create conditions that differ from undisturbed surrounding areas, driving a dynamic mosaic of microhabitats in the landscape differing in abiotic conditions and successional stage, thereby contributing to greater spatial and temporal heterogeneity within an environment.

Burrows appear to be more durable in the arid scrubland than the other sites (c. 30% of burrows had collapsed in the mesic grassland and the semi-arid savannah, but c. 10% in the arid scrubland), and this variation in burrow longevity (and the underlying differences in soils) may have contributed to the differences observed between the three habitats. The greater durability of burrows in the arid scrubland may reflect the high clay content of the soil^76^ (giving burrows a stronger structure) and the lower rainfall (which would slow degradation rates). In contrast, in the semi-arid savannah where burrows were created in sandy soil, burrows would probably collapse more frequently^77,78^. Although the semi-arid savannah site has a relatively low annual rainfall, even a moderate rainfall event appeared to trigger burrow collapse (pers. obs.). Therefore, in the semi-arid savannah, burrows are probably not long-lasting, and both biotic and abiotic conditions potentially converge with the undisturbed surrounding environment at a faster rate than at the other study sites. This suggests that the impacts of burrowing may be mediated by both soil type and rainfall. Indeed, contrary to initial predictions, rainfall may rather play a role as a physical force in the deterioration of burrows, where the combination of burrow durability and amount and intensity of rainfall will influence burrow longevity (see ref. ^20^ for a similar idea about engineered structures).

This research suggests that biogeomorphic ecosystem engineers do not necessarily have a stronger impact in abiotically-extreme environments (also see ref. ^79^), and that the impacts of ecosystem engineers may be site-specific^26^. Indeed, it was expected that the impact of burrowing would be greater in drylands, where productivity is low^8^ and resources are more limiting^51^. However, in this study, burrowing mammals had a greater effect on vegetation in the mesic environment than in the semi-arid environment. Furthermore, in the same way that the impacts of aardvark are inconsistent across environments, other ecosystem engineers may potentially also have inconsistent effects across their geographical distribution and potentially even within individual habitats. Site-specific impacts of ecosystem engineers may be related to environmental conditions, with the magnitude of the impact of ecosystem engineers possibly being stronger in environments where ecosystem engineers alter abiotic conditions to a greater extent than other abiotic and biotic processes and where biotic communities respond strongly to abiotic changes as a result of ecosystem engineering^80^. However, few studies have determined if the impacts of organisms with similar engineering roles vary across (or within) environments (although see ref. ^26,79,81,82^). As a result, there is a need to understand when ecosystem engineering is context-dependent or (relatively) context-independent, and therefore how predictable the ecological impacts of ecosystem engineering may be.

Ecosystem engineers may be influential species in conservation and restoration strategies, as numerous other species may be affected by the activities of a single ecosystem engineer^83,84^, especially where the species is an important biogeomorphic agent^85^. Due to ecosystem engineering potentially having site-specific impacts, management and conservation decision making involving ecosystem engineers are therefore not straightforward. As a result, to be able to use ecosystem engineers effectively as tools in conservation and restoration, we need to be able to understand and predict their impacts to some extent in different environments^4^. It therefore remains an important and relatively underexplored challenge to be able to predict variation in the impacts of ecosystem engineers across abiotic and biotic gradients.

## Supplementary information

Supplementary Information

## Data Availability

The datasets generated during and/or analysed during the current study are available from the corresponding author on reasonable request.
