# Supplementary material for: Testing for consistency in the impacts of a burrowing ecosystem engineer on soil and vegetation characteristics across biomes
Source: Sci Rep. 2019 Dec 18;9:19355. doi: 10.1038/s41598-019-55917-x (PMC6920383; doi:10.1038/s41598-019-55917-x)
Supplement: Supplementary file 1 — Supplementary Information [file 41598_2019_55917_MOESM1_ESM.pdf]

Supplementary Information

Testing for consistency in the impacts of a burrowing ecosystem engineer on soil and vegetation characteristics across biomes

M.A. Louw [a*#], N.S. Haussmann [b], P.C. le Roux [a]

a. *Department of Plant and Soil Sciences, University of Pretoria, Pretoria, Private Bag X20, Hatfield, 0028, South Africa*
b. *Department of Geography, Geoinformatics and Meteorology, University of Pretoria, Pretoria, Private Bag X20, Hatfield 0028, South Africa*

*Corresponding author: M.A. Louw

E-mail address: michelleannelouw87@gmail.com

#Current address: *Department of Biological Sciences, University of Cape Town, Private Bag X3, Rondebosch, 7701, South Africa*

Centre for Statistics in Ecology, Environment and Conservation, Department of Statistical Sciences, University of Cape Town, Private Bag X3, Rondebosch, 7701, South Africa

a b c

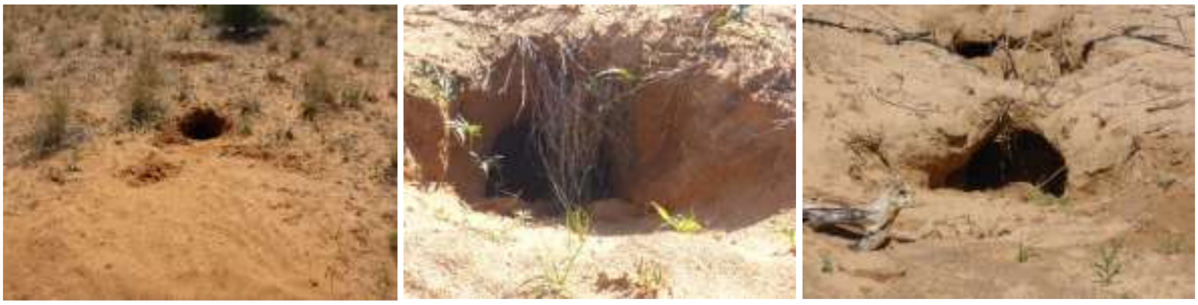

Fig. S.1: An example of different burrow age classes at the semi-arid savannah (Khamab Kalahari Reserve): a) a fresh burrow, b) an abandoned burrow, and c) a collapsed burrow.

Table S.1: The effects of burrowing mammals on vegetation cover and species richness at different study sites (see Fig. 2-4). Models also included burrows and associated microsites' locations as a random effect to account for the spatial clustering of sets of microsites. Detailed results are presented in Table S.2 – S.3.

Response variable	Study site	Fixed effects	Ranking of factor levels	Chi^2	df	p
Cover	Mesic grassland	Microsite	C > IA > BE	17.01	2	***
		Age	COL > AB > FR	5.62	2	p = 0.06
	Semi-arid savannah	Microsite	C > IA > BE	0.99	2	
		Age	COL > AB > FR	7.71	2	*
	Arid scrubland	Microsite	C > IA > BE	56.20	2	***
		Age	FR > AB	0.54	1	
Richness	Mesic grassland	Microsite	BE > C > IA	12.79	2	**
		Age	COL > AB > FR	6.02	2	*
		Microsite:Age		15.01	4	**
	Semi-arid savannah	Microsite	C > IA > BE	19.54	2	***
		Age	COL > AB > FR	18.87	2	***
	Arid scrubland	Microsite	C > IA > BE	38.49	2	***
		Age	AB > FR	0.06	1	

* $p < 0.05$, ** $p < 0.01$, *** $p < 0.001$

Table S.2: Pairwise post-hoc analyses of vegetation cover and species richness (see Table S.1 and Fig. 2-4). Models also included burrow location as a random effect to account for the spatial clustering of sets of microsites at each burrow. BE = "Burrow entrance", IA = "Impacted Area", C = "Control". Microsite type and burrow age were considered simultaneously as fixed effects in analyses, but are reported here in two separate tables for clarity (also see Table S.3). Post-hoc results are only reported where at least one pair of factor levels differed significantly.

Response variable	Study site	Microsites	Estimate	SE	z value	P
Vegetation cover	Mesic grassland	IA vs BE	0.64	0.38	1.68	
		C vs BE	1.95	0.47	4.12	***
		IA vs C	-1.31	0.48	-2.73	*
	Arid scrubland	IA vs BE	4.76	0.92	5.20	***
		C vs BE	6.29	0.93	6.74	***
		IA vs C	-1.53	0.32	-4.75	***
Species richness	Mesic grassland	IA vs BE	-0.36	0.11	-3.42	**
		C vs BE	-0.25	0.10	-2.43	*
		IA vs C	-0.11	0.11	-1.00	
	Semi-arid savannah	IA vs BE	0.04	0.20	0.20	
		C vs BE	0.64	0.17	3.72	***
		IA vs C	-0.60	0.17	-3.54	**
	Arid scrubland	IA vs BE	3.53	0.59	6.02	***
		C vs BE	3.63	0.59	6.20	***
		IA vs C	-0.10	0.14	-0.75	

* $p < 0.05$, ** $p < 0.01$, *** $p < 0.001$

Table S.3: Pairwise post-hoc analyses of vegetation cover and species richness (see Table S.1 and Fig. 2-4). Models also included burrow location as a random effect to account for the spatial clustering of sets of microsites at each burrow. FR = “Fresh”, AB = “Abandoned”, COL = “Collapsed”. Microsite type and burrow age were considered simultaneously as fixed effects in analyses, but are reported here in two separate tables for clarity (also see Table S.2). Post-hoc results are only reported where at least one pair of factor levels differed significantly.

Response variable	Study site	Age	Estimate	SE	z value	P
Vegetation cover	Mesic grassland	FR vs AB	-1.60	0.80	-2.01	
		FR vs COL	-1.96	0.83	-2.36	*
		COL vs AB	0.35	0.37	0.95	
	Semi-arid savannah	FR vs AB	-2.28	1.11	-2.05	
		FR vs COL	-2.90	1.06	-2.72	*
		COL vs AB	0.62	0.58	1.06	
Species richness	Mesic grassland	FR vs AB	-1.49	0.51	-2.92	**
		FR vs COL	-1.59	0.51	-3.10	**
		COL vs AB	0.11	0.12	0.91	
	Semi-arid savannah	FR vs AB	-0.79	0.21	-3.71	***
		FR vs COL	-0.79	0.20	-3.87	***
		COL vs AB	0.002	0.20	0.01	

* p < 0.05, ** p < 0.01, *** p < 0.001

Table S.4: Average cover (%) of common plant species (i.e. >3 occurrences) occurring at the different microsites (with the total number of microsites containing each of the vascular plant species in brackets) at the semi-arid savannah site. n = 71 for each microsite. Significant p-values indicate species where a species' occurrence across microsites differed significantly from what would be expected by chance.

Plant species	Burrow entrance	Impacted area	Control	p
Acanthosicyos naudinianus	0.2 (4)	0.3 (5)	1.1 (12)	*
Elephantorrhiza elephantina	0.0 (0)	0.4 (3)	0.3 (2)	
Gisekia pharnaceoides	0.4 (4)	0.0 (0)	0.2 (4)	
Ledebouria undulata	0.1 (1)	0.01 (1)	0.3 (5)	
Mollugo cerviana	0.03 (2)	0.03 (2)	0.1 (1)	
Ornithogalum seineri	0.0 (0)	0.03 (2)	0.1 (3)	
Schmidtia pappophoroides	0.4 (6)	0.6 (9)	0.6 (21)	**
Stipagrostis uniplumis	2.5 (22)	2.5 (19)	4.8 (30)	
Talinum crispatulum	0.01 (1)	0.2 (2)	0.1 (2)	
Tribulus zeyheri	0.8 (5)	0.5 (2)	0.0 (0)	

* $p < 0.05$, ** $p < 0.01$, *** $p < 0.001$

Table S.5: Average cover (%) of common plant species (i.e. >3 occurrences) occurring at the different microsites (with the total number of microsites containing each of the vascular plant species in brackets) at the mesic grassland site. Plant species shown in bold are unique to a specific microsite. n = 60 for each microsite. Significant p-values indicate species where a species' occurrence across microsites differed significantly from what would be expected by chance.

Plant species	Burrow entrance	Impacted area	Control	p
Acalypha angustata	1.3 (6)	1.4 (8)	1.0 (8)	
Aristida congesta	0.1 (2)	0.1 (1)	0.2 (1)	
Bidens bipinnata	0.4 (5)	0.1 (4)	0.1 (2)	
Bidens pilosa	0.8 (5)	0.5 (2)	0.02 (1)	
Campuloclinium macrocephalum	0.9 (12)	0.4 (5)	0.4 (5)	
Cheilanthes viridis	**6.4 (13)**	0.0 (0)	0.0 (0)	***
Commelina africana	0.3 (3)	0.2 (3)	0.2 (1)	
Conyza bonariensis	1.3 (9)	0.5 (5)	0.2 (2)	
Conyza podocephala	0.6 (6)	0.5 (7)	1.0 (7)	
Conyza sp1	0.7 (6)	0.1 (2)	0.0 (0)	*
Cymbopogon excavatus	0.4 (3)	1.2 (3)	3.1 (9)	
Cynodon dactylon	8.1 (26)	12.3 (27)	5.3 (16)	
Eragrostis chloromelas	2.4 (15)	8.7 (25)	17.8 (36)	**
Eragrostis curvula	0.2 (1)	2.4 (5)	1.4 (4)	
Eragrostis lehmanniana	3.9 (17)	11.5 (25)	13.0 (21)	
Eragrostis plana	0.1 (2)	0.3 (1)	0.1 (1)	
Eragrostis tef	0.1 (1)	0.4 (3)	4.2 (7)	
Helichrysum rugulosum	1.2 (6)	2.3 (10)	2.9 (12)	
Hermannia depressa	0.02 (1)	0.4 (2)	0.1 (1)	
Heteropogon contortus	0.3 (3)	1.0 (1)	1.8 (5)	
Hilliardiella oligocephala	1.8 (11)	1.0 (6)	0.7 (8)	
Hyparrhenia hirta	1.4 (6)	0.8 (7)	2.0 (6)	
Hypoxis iridifolia	0.0 (0)	0.3 (2)	0.4 (3)	
Hypoxis sp3	0.7 (3)	0.4 (2)	0.3 (3)	
Morpho *sp37*	0.7 (4)	0.3 (1)	0.0 (0)	
Morpho *sp48*	1.1 (4)	1.4 (5)	1.2 (4)	
Grass sp1	1.1 (3)	1.0 (1)	4.7 (4)	
Oenothera rosea	0.4 (4)	0.1 (2)	0.2 (5)	
Oenothera sp	0.4 (4)	0.1 (1)	0.0 (0)	
Oxalis corniculata	1.3 (23)	0.3 (3)	0.3 (6)	***
Panicum natalense	0.5 (3)	1.4 (5)	2.1 (4)	
Pelargonium luridum	0.1 (2)	0.02 (1)	0.1 (1)	
Pellaea calomelanos	**0.4 (4)**	0.0 (0)	0.0 (0)	*
Pollichia campestris	1.2 (6)	0.0 (0)	0.02 (1)	**
Schkuhria pinnata	0.4 (4)	0.7 (4)	0.3 (2)	
Senecio inornatus	0.5 (7)	0.7 (6)	1.1 (12)	
Senecio sp2	0.5 (9)	0.02 (1)	0.1 (2)	**
Setaria sphacelata1	0.02 (1)	0.2 (3)	0.1 (1)	

Solanum tomentosum	0.3 (5)	0.2 (6)	0.2 (5)	
Tagetes minuta	1.1 (13)	0.2 (4)	0.03 (2)	**
Teucrium trifidum	0.3 (4)	0.02 (1)	0.0 (0)	
Themeda triandra	0.3 (1)	1.6 (4)	0.7 (2)	
Thesium utile	0.1 (2)	0.3 (2)	0.0 (0)	
Urochloa panicoides	0.1 (2)	0.3 (1)	0.02 (1)	
Verbena bonariensis	5.0 (15)	1.0 (8)	0.5 (3)	**
Verbena brasiliensis	0.6 (5)	0.5 (3)	0.5 (4)	

* $p < 0.05$, ** $p < 0.01$, *** $p < 0.001$

Table S.6: Average cover (%) of common plant species (i.e. >3 occurrences) occurring at the impacted area and control microsites (with the total number of microsites containing each of the vascular plant species in brackets) at the arid scrubland site. n = 11 for each microsite. No species differed significantly in frequency of occurrence between the two microsites.

Plant species	Impacted area	Control
Atriplex lindleyi ssp. *inflata*	3.3 (4)	1.4 (6)
Augea capensis	2.2 (6)	3.9 (9)
Crassula subaphylla	0.5 (5)	0.3 (2)
Drosanthemum hispidum	0.9 (7)	1.3 (6)
Drosanthemum praecultum	1.0 (2)	0.6 (6)
Galenia fruticosa	0.7 (7)	1.5 (9)
Malephora lutea	15.6 (10)	33.2 (11)
Osteospermum sinuatum	0.6 (5)	2.5 (9)
Phyllobolus nitidus	0.6 (5)	2.8 (8)
Phyllobolus splendens	1.6 (7)	1.8 (6)
Psilocaulon junceum	17.6 (10)	31.2 (11)
Pteronia pallens	7.1 (11)	7.5 (10)
Ruschia spinosa	2.5 (9)	3.0 (9)

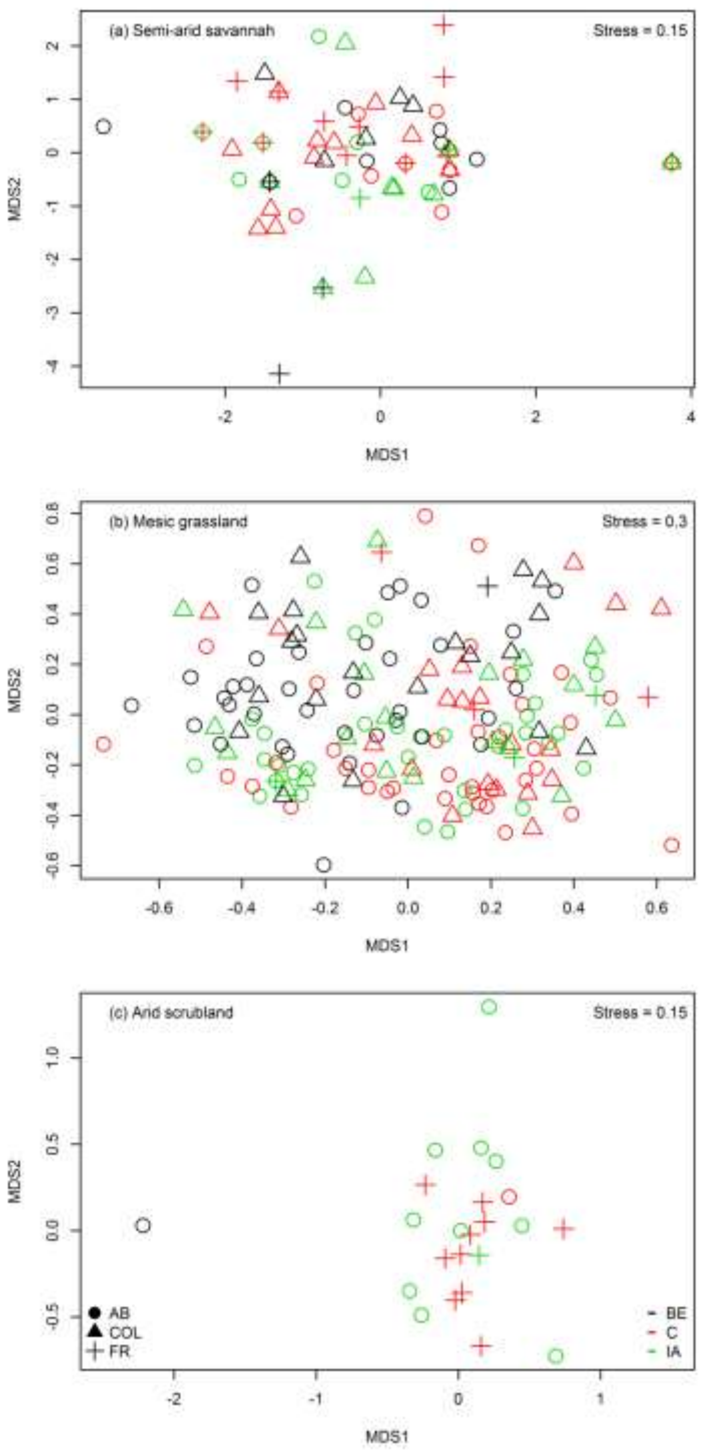

Fig. S.2: Vegetation composition at the different study sites. Colour indicates microsites (i.e. black = burrow entrance, red = control, green = impacted area) and symbols indicates burrow age (i.e. ○ = abandoned, △ = collapsed, + = fresh). BE = “Burrow entrance”, IA = “Impacted area”, C = “Control”. AB = “Abandoned”, COL = “Collapsed”, FR = “Fresh”

Table S.7: Overall model results of the effects of burrowing mammals on soil temperature, moisture and compaction at different study sites (see Fig. 5-7). Models also included burrow location as a random effect to account for the spatial clustering of sets of microsites at each burrow. Detailed results presented Table S.8 – S.9).

Response variable	Study site	Fixed effects	Chi^2	Df	p
Temperature	Mesic grassland	Microsite	135.73	2	***
		Time	103.19	1	***
		Age	2.82	2	
		Microsite:Time	23.80	2	***
	Semi-arid savannah	Microsite	230.72	2	***
		Time	75.66	1	***
		Age	0.94	2	
		Microsite:Time	21.57	2	***
	Arid scrubland	Microsite	2.46	2	
		Time	4.23	1	*
		Age	0.04	1	
		Microsite:Time	21.30	2	***
Moisture	Mesic grassland	Microsite	51.31	2	***
		Age	2.84	2	
	Semi-arid savannah	Microsite	23.03	2	***
		Age	3.39	2	
	Arid scrubland	Microsite	4.34	2	
		Age	2.82	1	
Compaction	Mesic grassland	Microsite	25.62	2	***
		Age	2.11	2	
	Semi-arid savannah	Microsite	64.85	2	***
		Age	2.97	2	
	Arid scrubland	Microsite	2.40	2	
		Age	0.12	1	

* $p < 0.05$, ** $p < 0.01$, *** $p < 0.001$

Table S.8: Pairwise post-hoc analyses of soil temperature (see Table S.7 and Fig. 5). Models also included burrow location as a random effect to account for the spatial clustering of sets of microsites at each burrow. BE = "Burrow entrance", IA = "Impacted Area", C = "Control". Post-hoc results are only reported where at least one pair of factor levels differed significantly.

Study site	Microsites	Fixed effects	Chi^2	df	P
Mesic grassland	IA vs BE	Microsite	113.96	1	***
		Time	83.42	1	***
		Age	1.48	2	
		Microsite:Time	21.27	1	***
	C vs BE	Microsite	155.66	1	***
		Time	60.38	1	***
		Age	2.93	2	
		Microsite:Time	10.41	1	**
	IA vs C	Microsite	6.39	1	*
		Time	104.28	1	***
		Age	2.72	2	
		Microsite:Time	5.17	1	*
Semi-arid savannah	IA vs BE	Microsite	140.29	1	***
		Time	61.26	1	***
		Age	1.35	2	
		Microsite:Time	13.58	1	***
	C vs BE	Microsite	163.02	1	***
		Time	77.89	1	***
		Age	1.51	2	
		Microsite:Time	14.83	1	***
	IA vs C	Microsite	1.50	1	
		Time	72.06	1	***
		Age	0.26	2	
		Microsite:Time	0.05	1	
Arid scrubland	IA vs BE	Microsite	0.72	1	
		Time	3.98	1	*
		Age	0.10	1	
		Microsite:Time	19.14	1	***
	C vs BE	Microsite	1.58	1	
		Time	3.36	1	
		Age	0.06	1	
		Microsite:Time	7.1	1	**
	IA vs C	Microsite	1.21	1	
		Time	5.27	1	*
		Age	0.002	1	
		Microsite:Time	2.96	1	

* $p < 0.05$, ** $p < 0.01$, *** $p < 0.001$

Table S.9: Pairwise post-hoc analyses of soil moisture and soil compaction (see Table S.7 and Figures 6-7). Models also included burrow location as a random effect to account for the spatial clustering of sets of microsites at each burrow. BE = “Burrow entrance”, IA = “Impacted Area”, C = “Control”. Microsite type and burrow age were considered simultaneously as fixed effects in analyses but are reported here in two separate tables for clarity. Post-hoc results are only reported where at least one pair of factor levels differed significantly.

Response variable	Study site	Microsites	Estimate	SE	z value	P
Moisture	Mesic grassland	IA vs BE	2.17	0.62	3.51	**
		C vs BE	4.43	0.62	7.16	***
		IA vs C	-2.26	0.62	-3.65	***
	Semi-arid savannah	IA vs BE	-0.11	0.25	-0.43	
		C vs BE	-1.08	0.25	-4.36	***
		IA vs C	0.97	0.25	3.92	***
Compaction	Mesic grassland	IA vs BE	3.63	1.04	3.47	**
		C vs BE	4.79	1.05	4.57	***
		IA vs C	-1.17	0.39	-3.02	**
	Semi-arid savannah	IA vs BE	1.51	0.19	8.05	***
		C vs BE	1.15	0.19	5.91	***
		IA vs C	0.36	0.13	2.72	*

* p < 0.05, ** p < 0.01, *** p < 0.001
